# Determination of Ochratoxin A in Rye and Rye-Based Products by Fluorescence Polarization Immunoassay

**DOI:** 10.3390/toxins9100305

**Published:** 2017-09-26

**Authors:** Vincenzo Lippolis, Anna C. R. Porricelli, Marina Cortese, Michele Suman, Sandro Zanardi, Michelangelo Pascale

**Affiliations:** 1 Institute of Sciences of Food Production (ISPA), National Research Council of Italy (CNR), Bari 70126, Italy; chiara.porricelli@ispa.cnr.it (A.C.R.P.); marina.cortese@ispa.cnr.it (M.C.); michelangelo.pascale@ispa.cnr.it (M.P.); 2 Barilla Advanced Laboratory Research, Parma 43122, Italy; michele.suman@barilla.com (M.S.); sandro.zanardi@barilla.com (S.Z.)

**Keywords:** fluorescence polarization immunoassay, ochratoxin A, rye, rye-based products, validation study

## Abstract

A rapid fluorescence polarization immunoassay (FPIA) was optimized and validated for the determination of ochratoxin A (OTA) in rye and rye crispbread. Samples were extracted with a mixture of acetonitrile/water (60:40, *v*/*v*) and purified by SPE-aminopropyl column clean-up before performing the FPIA. Overall mean recoveries were 86 and 95% for spiked rye and rye crispbread with relative standard deviations lower than 6%. Limits of detection (LOD) of the optimized FPIA was 0.6 μg/kg for rye and rye crispbread, respectively. Good correlations (r > 0.977) were observed between OTA contents in contaminated samples obtained by FPIA and high-performance liquid chromatography (HPLC) with immunoaffinity cleanup used as reference method. Furthermore, single laboratory validation and small-scale collaborative trials were carried out for the determination of OTA in rye according to Regulation 519/2014/EU laying down procedures for the validation of screening methods. The precision profile of the method, cut-off level and rate of false suspect results confirm the satisfactory analytical performances of assay as a screening method. These findings show that the optimized FPIA is suitable for high-throughput screening, and permits reliable quantitative determination of OTA in rye and rye crispbread at levels that fall below the EU regulatory limits.

## 1. Introduction

Several *Penicillium* and *Aspergillus* species growing in different agricultural commodities in the field or during storage can produce ochratoxin A (OTA) [1], a mycotoxin that has recently received a special focus due to its toxic effects. Indeed, OTA has multiple toxic effects that are a real menace to the health of humans and animals. Many studies have indicated that this mycotoxin shows nephrotoxic, hepatotoxic, embryotoxic, teratogenic, neurotoxic, immunotoxic, genotoxic, and carcinogenic effects on several species [2]. Furthermore, OTA is associated with the aetiology of Balkan Endemic Nephropathy (BEN) and in Chronic Interstitial Nephropathy [3]. The International Agency for Research on Cancer (IARC) has included OTA in the group 2B classifying it as a possible human carcinogen due to its capacity to induce cancer in animals [4]. OTA has been extensively reported to contaminate a wide variety of foods and beverages such as cereals and derived products, coffee, spices, grapes, beer and wine at global level [5,6,7,8]. However, a recent study on the assessment of OTA intake by European consumers showed that cereals are the most important dietary source of OTA [9]. According to recent reports on food incidence of OTA, cereals and their cereal-based products contribute similarly to OTA exposure in other part of the world [7]. On the basis of data occurrence in Europe and from Rapid Alert System for Food and Feed (RASFF) of the European Union, OTA occurs in wheat, oat, corn, barley, rye, rice, millet and derived products [9,10]. Among all cereals, rye shows the highest level of contamination, with 50% of positive samples, mainly found in North Europe [9]. The major producers of rye in 2014 were Germany, Poland, Russia, Belarus and Denmark, and its average annual consumption ranges from over 22 kg/capita in Poland, Belarus and Estonia to 14–18 kg/capita in Lithuania, Denmark, Finland and Latvia [11]. Moreover, in these countries, rye plays an important role in the fibre intake, contributing almost 40% of dietary fibre, due to the high consumption of wholemeal rye and rye-based products, such as rye bread, crispbread and rye flakes [12]. The increased awareness of consumers of the important relationship between diet and well-being has raised their interest toward rye and rye-based products [13]. Considering the important role of cereals in human diet at global level and to protect the health of consumers from the risk of OTA exposure, the European Commission has fixed maximum permitted levels for this toxin in unprocessed cereals (5 μg/kg) and derived products (3 μg/kg) [14].

During recent decades, a large number of analytical methods have been developed and validated for OTA determination, alone or in combination with other mycotoxins, in order to meet the legislative requirements [15,16]. Analytical methods based on high-performance liquid chromatography (HPLC) coupled with fluorescent detection after immunoaffinity column (IAC) cleanup are the most commonly used approaches for OTA determination in cereals and derived products [17]. Furthermore, some HPLC methods based on IAC cleanup have been successively adopted by AOAC and CEN, as official or standard methods, for OTA determination in barley and in cereal-based foods for infants and young children [18,19,20]. Additionally, other analytical methods have been developed for simultaneous determination of mycotoxins, including OTA, in various matrices based on high performance liquid chromatography coupled with mass spectrometry (LC-MS/MS) [21]. Despite these analytical methods allowing OTA determination with high sensitivity and accuracy, they are expensive and time-consuming, and require qualified personnel, and thus are not suitable for detection in large numbers of samples. For this reason, the development of simple, rapid, cheap and reliable analytical methods for OTA monitoring is resulting in the emergence of screening tools. A large number of rapid methods for mycotoxin analysis in food and feed have been developed, such as enzyme-linked immunosorbent assay, lateral flow devices or dipstick, flow-through immunoassay, optical and electrochemical biosensors and, more recently, aptamer-based methods [22,23,24,25,26,27]. Among them, fluorescent polarization immunoassay (FPIA), a promising screening method and its application, has been recently reviewed by Lippolis and Maragos [28]. Fluorescent polarization (FP) is a powerful technique that permits the study of the molecular interaction by monitoring the rate of a fluorophore rotation in solution. In particular, FPIA is a homogeneous immunoassay based on the competition between the analyte and its fluorescent derived (tracer) for specific antibody-binding sites. The amount of free analyte present in the sample is inversely related to the amount of bound tracer; thus, the polarization value is inversely proportional to the concentration of analyte in solution [28]. Several FPIA methods have been developed as screening tools for the determination of major mycotoxins (i.e., aflatoxins, OTA, zearalenone, fumonisins, deoxynivalenol and T-2 and HT-2 toxins) [29,30,31,32,33,34,35,36,37,38,39]. In particular, some FPIAs have been developed for OTA determination in unprocessed cereals such as barley, wheat and rice [40,41,42,43]. To date, no FPIA for the determination of OTA in rye and rye-based products have been developed and validated. For this reason, the aim of this study was to assess the applicability of an FPIA, previously described by Lippolis el al. for the determination of OTA in wheat [43], to the determination of OTA in rye and rye-based products (e.g., rye crispbread). A solid-phase extraction (SPE) cleanup procedure was optimized to remove matrix interferences for reliable quantitative OTA determination at levels below the maximum permitted levels fixed by the EU [14]. The method has been validated in-house by using artificially contaminated samples for comparison with a reference method based on HPLC and immunoaffinity column cleanup of extracts. Furthermore, harmonized guidelines have been established by the European Commission in the Regulation No 519/2014/EU for the evaluation of fitness of purpose performance parameters of screening methods for the detection of mycotoxins in food [44]. Recently, practical applications of EU guidelines have been reported for the determination of deoxynivalenol in wheat and wheat dust [45,46], and for the simultaneous determination of *Fusarium* toxins in wheat and maize by immunoassays [47]. In this paper, validation of FPIA for the determination of OTA in rye has been carried out through single-laboratory validation and a small collaborative trial according to EU guidelines [44]. The fitness of purpose of the FPIA was evaluated by calculating the precision profile of the method and setting the screening target concentration (STC) for false suspect rate and cut-off level to the EU’s maximum permitted level of OTA in rye [14].

## 2. Results and Discussion

### 2.1. Matrix Effect and SPE Cleanup

**Figure 1:**
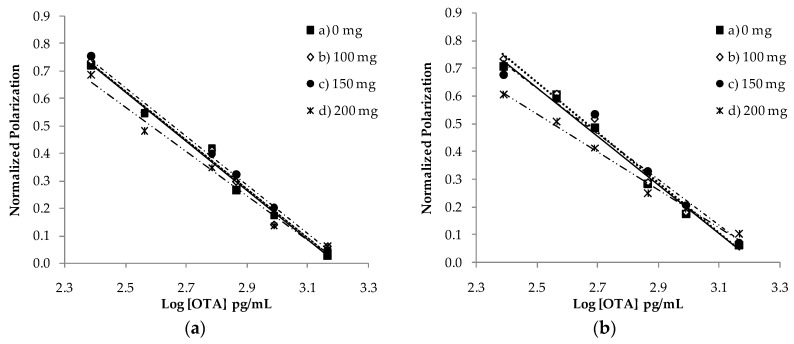
Calibration curves (concentration range from 0.25 to 1.5 ng/mL) obtained with OTA standard solutions (*black square*) and spiked diluted extracts of rye (**a**) and rye crispbread (**b**) by analysing 100 mg (*white diamond*), 150 mg (*black circle*) and 200 mg (*asterisk*) of matrix equivalent.

The evaluation of the matrix effect on the FPIA was performed by using spiked extracts of rye and rye crispbread at different OTA levels, in the range 2.5–75 ng/mL, and analysing different amounts of matrix equivalent of 1, 2.5 and 5 mg. The effect due to the matrix was observed when analysing 5 mg of matrix equivalent for both matrices tested. Under these conditions, the sensitivity of the immunoassay was very low, with a limit of quantification (LOQ) of 100 μg/kg. In order to increase the assay sensitivity, and to avoid matrix interferences, a rapid SPE cleanup of the extracts was optimized by using an aminopropyl silica sorbent recently used as solid-phase material in the purification of wheat for OTA determination by FPIA [43]. In particular, filtered extracts of the rye and the rye crispbread samples were purified through a SPE Bond Elut NH_2_ column. After the SPE cleanup, without a conditioning or washing step, OTA was eluted with a mixture of methanol/water/acetic acid 50:50:1 (*v*/*v*/*v*). The elution was performed in 1.75 mL for rye and 1.50 mL for rye crispbread samples; 1 mL was collected after discharging the first aliquots (0.75 mL and 0.50 mL, respectively). To estimate the matrix effect on the FP measurements after SPE cleanup, the regression line obtained with OTA standard solutions in the range 0.25–1.5 ng/mL was compared with regression lines performed by adding spiked purified extracts of rye and rye crispbread uncontaminated samples, as illustrated in Figure 1. In particular, spiked purified extracts of rye and rye crispbread were analysed by using 100, 150 and 200 mg of matrix equivalent. The parallelism and position statistical tests showed no significant differences between slopes (*t_calc_* < 2.306; *p*-value > 0.05) and positions (*t_calc_* < 2.262; *p*-value > 0.05) of the regression lines obtained with OTA standard solutions in buffer and those obtained with spiked purified extracts of rye and rye crispbread using 150 mg of matrix equivalent. These results showed that the rapid SPE cleanup procedure significantly decreases the matrix effect on the FPIA. 

### 2.2. In-House Validation of the Optimized FPIA as Quantitative Method

A limit of detection (LOD) of 0.09 ng/mL (equivalent to 0.6 μg/kg in samples) was calculated for the optimized FPIA (analysing 150 mg of matrix equivalent) for rye and rye crispbread samples. These results showed that the LOD obtained was far below the maximum permitted levels established by the EU for OTA in unprocessed cereals (i.e., 5 μg/kg) and derived products (i.e., 3 μg/kg) [14]. Furthermore, the limit of quantification (LOQ) of the FPIA for rye and rye crispbread was 1.7 μg/kg. In the case of rye, the LOQ value fulfils performance criteria fixed by CEN for the acceptance of LOQ of single-laboratory validated methods for mycotoxin determination [48]. Indeed, for contamination levels <100 μg/kg, the LOQ value should be less than or equal to the 2/5 × maximum legal limit of the toxin. Results of recovery experiments in terms of accuracy and repeatability for both matrices at levels 2, 5, 8 μg/kg are reported in Table 1.

**Figure 2:**
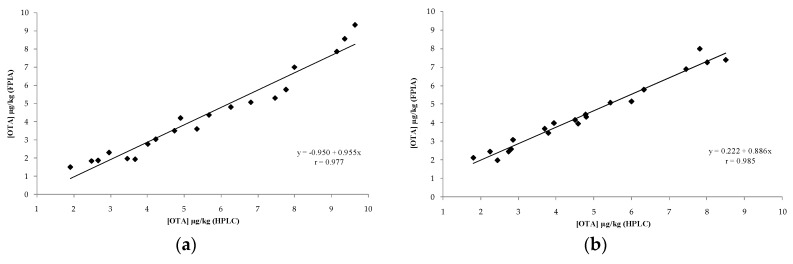
Comparison of OTA contents in artificially contaminated samples of rye (**a**) and rye crispbread (**b**) analysed by FPIA and HPLC reference method (data corrected for average recoveries).

Overall average recoveries for FPIA were 86 and 95% for rye and for rye crispbread, respectively, with relative standard deviations ranging from 2 to 6%. While average recoveries obtained for the HPLC reference method were 99 and 94% for rye and for rye crispbread, respectively, with relative standard deviations ≤ 6%. The values of accuracy and precision obtained for the optimized FPIA fulfil the criteria of acceptability of an analytical method for OTA determination fixed by the European Commission [49]. In addition, a comparative analysis of a total of 30 rye and rye crispbread samples, of which 20 were spiked samples with OTA contaminations in the range 2–9.5 μg/kg and were 10 blank samples (uncontaminated samples), was carried out by both FPIA and HPLC method. Good correlations were found for the tested matrices between OTA concentrations obtained by FPIA and those obtained by the HPLC reference method, with coefficients of correlation (*r*) of 0.977 and 0.985 in rye and in rye crispbread, respectively (Figure 2). The linear regression fits were [OTA by FPIA] = −0.950 + 0.955 [OTA by HPLC] for rye and [OTA by FPIA] = +0.222 + 0.886 [OTA by HPLC] for rye crispbread (data corrected for average recoveries). These findings confirmed the good performance of the optimized FPIA in terms of accuracy and precision. Furthermore, no false positive result was observed in the analysis of blank samples by FPIA. 

### 2.3. Evaluation of Analytical Performances of the Optimized FPIA According to Regulation 519/2014/EU

The analytical performance profile for the FPIA for the determination of OTA in rye was integrated according to the validation guidelines for mycotoxin screening methods reported in Regulation 519/2014/EU. Single-laboratory validation experiments performed over 5 different days and a small-scale collaborative trial, involving two laboratories were carried out. The aim of this validation was to demonstrate the fitness of the developed FPIA for the purpose of assessing rye sample compliance with EU maximum permitted levels. This was done by determination of the precision profile of the method, cut-off level and the rate of false suspect results. Results of the statistical assessment of the single-laboratory validation and small-scale collaborative trial are shown in Table 2.

**Figure 3:**
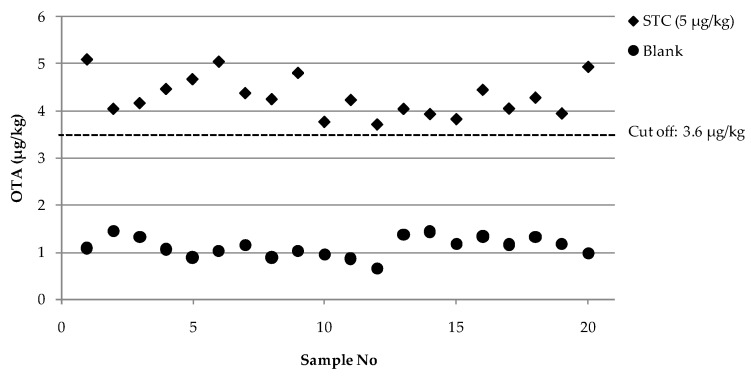
OTA content (μg/kg) by FPIA for 20 artificially contaminated rye samples at STC (5 μg/kg) and for 20 blank rye samples analysed in 5 different days under repeatability conditions. The cut-off value was calculated by using the results of the samples containing OTA at target level.

Concerning the single-laboratory validation, mean values of the test response for OTA were 4.3 and 1.1 μg/kg, with relative standard deviation under repeatability conditions (RSDr) of 10 and 13% and a relative standard deviation under within-reproducibility conditions (RSD_RI_), i.e., intermediate precision, of 10 and 19% for artificially contaminated samples at screening target concentration (STC) of OTA and uncontaminated samples (blank), respectively. Taking into account the mean value and standard deviation of STC (SD_STC_), estimated by analysis of variance (nested ANOVA, *p*-value = 0.05), the cut-off level and the rate of suspect results were calculated as reported in the Materials and Methods section. In particular, the cut-off level result was 3.6 μg/kg and the rate of suspect results for blank samples was less than 0.1%. No significant difference was observed in the determination of OTA content over 5 different days at STC (*p*-value = 0.402). The overall intermediate precision data, with results equal to or less than 22%, can be considered acceptable, taking into account the typical performance profile of immunoassays when applied for screening purposes. Moreover, Figure 3 shows a graphical presentation of the results, reporting the OTA content determined in 20 artificially contaminated rye samples at STC and in 20 blank rye samples, analysed over 5 different days with the calculated cut-off level. The plot highlights the ability of the method to discriminate between blank and artificially contaminated samples at STC providing two well defined groups of results. This aspect was also mirrored by the negligible rate of suspect results (i.e., less than 0.1%). These results indicate FPIA performances suitable for small scale collaborative trials.

As well as the single-laboratory validation, a small-scale collaborative trial was carried out according to the inter-laboratory validation procedure trials as reported in Regulation 519/2014/EU [44]. In particular, the determined OTA mean values were 4.8 and 0.9 μg/kg, with RSD_r_ of 9 and 16% and RSD_R_ of 10 and 22% for artificially contaminated samples at STC of OTA and for blank samples, respectively. The calculated cut-off level result was 4.0 μg/kg, with a rate of false suspect results for blank samples below 0.1%. Results obtained in the collaborative trial confirmed the analytical performances obtained by the single-laboratory validation and the small-scale collaborative trial in terms of the calculated cut-off level and rate of false suspect results. The overall results confirmed the applicability of the FPIA for the determination of OTA in rye to discriminate samples contaminated at the EU maximum permitted level of OTA in unprocessed cereals from uncontaminated samples.

## 3. Conclusions

An accurate FPIA, previously described for the determination of OTA in wheat, was applied to the analysis of rye and rye crispbread. A rapid SPE cleanup was optimized for both matrices to remove matrix interferences, allowing a very high sensitivity to be reached, with a LOD far below the European regulatory limits for OTA in cereals and derived products. Despite the purification step, the optimized protocol was rapid and easy to perform, permitting the FPIA to be carried out in a total time of less than 30 min. The optimized FPIA showed analytical performances, in terms of accuracy and precision, that fulfilled the criteria for acceptability of an analytical method for the determination of OTA established by the European Union [49]. In addition, good correlations were observed between OTA contents in contaminated samples obtained by both FPIA and HPLC with immunoaffinity cleanup used as reference method. Furthermore, in line with recent harmonized guidelines for the validation of screening methods, an experimental protocol for single-laboratory validation and small-scale collaborative trial has been defined and applied to the determination of OTA in rye by FPIA according to Regulation 519/2014/EU. The satisfactory analytical performances, in terms of precision under repeatability, within laboratory reproducibility (intermediate precision) and inter-laboratory reproducibility conditions and the cut-off level confirmed the applicability of the proposed assay as a screening method for assessing OTA content in rye at regulatory levels, with a false positive rate of less than 0.1%. Moreover, the optimized assay is low in cost, uses a portable instrument, can be automated, and does not require a high level of technical skills. These findings indicate that the proposed FPIA is appropriate for high-throughput screening, as well as for quantitative OTA determination in rye and rye crispbread, and represents an alternative approach to more expensive and time-consuming LC methods.

## 4. Materials and Methods 

### 4.1. Reagents and Chemicals

OTA, sodium tetraborate decahydrate (B_4_Na_2_O_7_ × 10 H_2_O), TWEEN^®^ 20, sodium azide (NaN_3_) and ovalbumin (OVA) were obtained from Sigma-Aldrich (Milan, Italy). MAb clone 5E2 (Order No. 201052) was provided by Softflow Biotechnology (Pécs, Hungary). Glass culture tubes (10 × 75 mm) were supplied by VWR International S.R.L. (Milan, Italy). Glass microfiber filters (Whatman GF/A) and paper filters (Whatman N. 4) were purchased from Whatman (Maidstone, UK). Solid phase extraction columns Bond Elut NH_2_ (500 mg, 3 mL) were purchased by Agilent Technologies (Santa Clara, CA, USA). OchraTest™ immunoaffinity columns were provided by VICAM, a Water Business (Milford, MA, USA). All other chemicals and solvents were reagent grade or HPLC grade and were obtained from Sigma-Aldrich (Milan, Italy). Ultrapure water was produced by a Waters Milli-Q system (Waters, Milford, MA, USA).

### 4.2. OTA Standard and Immunoreagent Solutions

A stock solution of OTA was prepared at a concentration of 1 mg/mL in toluene:acetic acid 99:1 (*v*/*v*). A standard OTA solution, at the concentration of 10 μg/mL, was prepared in methanol and then spectrophotometrically tested at λ = 332 nm (ε = 6330 cm^2^/mmol). An additional OTA solution, at concentration of 250 ng/mL, was prepared in methanol for the preparation of standard solutions for FPIA and HPLC calibrations and for spiking purposes in recovery experiments. Concerning the standard solutions for HPLC calibration, an aliquot of the diluted OTA solution (250 ng/mL) in methanol was dried under a stream of nitrogen and then dissolved in a mixture acetonitrile/water/acetic acid 99:99:2 (*v*/*v*/*v*). OTA standard solutions for FP calibration were prepared diluting opportune volumes of OTA spiking solution (250 ng/mL) in methanol. FP measurements were carried out by using OTA-fluorescein tracer (OTA-FL), prepared according to Lippolis et al. [43]. An aliquot of the tracer stock solution was diluted at ratio of 1:10,000 (*v*/*v)* in methanol for the preparation of the tracer working solution. Daily, an aliquot of OTA monoclonal antibody clone 5E2 was diluted at a rate of 1:200 *(v*/*v)* in borate buffer, pH = 8.5 with 0.1% sodium azide and 0.1% of ovoalbumin (BB-OVA) in order to prepare the antibody working solution.

### 4.3. Sample Preparation

Rye and rye crispbread samples were collected by suppliers and local markets located in northern European countries. Samples were milled by an Ultra Centrifugal Mill ZM 200 (Retsch Technology GmbH, Haan, Germany) laboratory mill (sieve of 500 μm). Sample extractions were carried out in compliance with the procedure reported by Lippolis et al. [43], with minor modifications. An aliquot of milled samples (25 g) was extracted with 100 mL of acetonitrile/water 60:40 (*v*/*v*) into a blender jar by blending with a Steril Mixer 12 blender (International PBI, Milan, Italy), at high speed for 3 min. After filtration with filter paper (Whatman N. 4), extracts were analysed by FPIA or by HPLC analysis as described below. In order to assess the presence of a matrix effect on the FPIA when performed without a cleanup procedure, filtered extracts of blank rye and rye crispbread samples were diluted with water in a ratio 1:5 (*v*/*v*), filtered through a glass microfiber filter (Whatman GF/A) and spiked at different OTA levels (in the range 2.5–75 ng/mL). These spiked solutions were analysed by FPIA at different amounts of matrix equivalent of 1, 2.5 and 5 mg. Rye and rye crispbread blank samples used in this study were selected by HPLC reference method as reported below.

### 4.4. SPE Cleanup

Aliquots of filtered extracts, 6 mL and 2 mL for rye and rye crispbread extracts respectively, were loaded on a SPE Bond Elut NH_2_ column keeping a flow rate of about one drop per second. OTA was eluted with 1.75 mL and 1.50 mL of methanol/water/acetic acid 50:50:1 (*v*/*v*/*v*) for rye and rye crispbread samples, respectively. The first aliquot (0.75 mL for rye and 0.50 mL for rye crispbread sample) was discharged, and the remaining portion (1 mL) was collected in a silanized vial at a flow rate of one drop per second and analysed by FPIA. SPE column was dried during each step, and no column conditioning or washing step was performed. The matrix effect of the purified extract was evaluated by spiking it at different OTA levels (in the range 1.88–22.5 ng/mL for rye and 0.63–7.5 ng/mL for rye crispbread), and analysing by FPIA.

### 4.5. FPIA Analysis

Antibody cross-reactivity was previously tested against ochratoxin B (OTB) and other mycotoxins commonly occurring in wheat showing high specificity for OTA [43]. FPIA analyses were performed by the Sentry^®^ 100 portable system (Diachemix Corporation, Milwaukee, WI, USA), a manual single-well instrument using 10 × 75-mm glass culture tubes (VWR International S.R.L., Milan, Italy) and excitation (λ_ex_) and emission (λ_em_) wavelengths of 485 and 535 nm, respectively. The FPIAs were carried out in accordance with the method proposed by Lippolis et al. [43], with minor modifications. FP analyses were carried out by adding 100 μL of antibody working solution in the test tube with 50 μL of OTA standard solution or 100 μL of purified rye extract or 300 μL of purified rye crispbread extract (i.e., 150 mg of matrix) and borate buffer, pH = 8.5 with 0.1% sodium azide (BB-A) up to 1000 μL. The test solution was mixed and then placed in the instrument for the measurement of the signal that was used as the blank of determination. After removing the test tube from the reader, 25 μL of tracer working solution (OTA-FL) was added in the test solution and mixed for an incubation time of 5 min. The test solution was replaced in the reader and the polarization value was measured. In order to normalize the polarization value (expressed in mP, i.e., millipolarization units) to fit the range 0–1, the equation Y_obs_ = (mP_obs_ − mP_0_)/(mP_1_ − mP_0_) was used, where mP_obs_, mP_0_ and mP_1_ are the polarization of the test solution, of an antibody-free control solution and of a toxin-free control solution, respectively, and Y_obs_ is the normalized result for the test solution [28]. The content of OTA in the sample extracts was calculated by measuring normalized polarization values and using the FP calibration curves in OTA concentration range 0.25–1.5 ng/mL.

### 4.6. HPLC Analysis

OTA analyses of rye and rye crispbread samples were performed according to the reference method for the determination of OTA in barley (AOAC Official Method 2000.03) [18], with minor modifications. Aliquots of filtered extracts (10 mL) were diluted with water in a ratio 1:4 and filtered using a glass microfiber filter. Diluted filtered extracts aliquots (10 mL, corresponding to 0.5 g sample) were loaded onto the immunoaffinity column keeping a flow rate of about one drop per second. IAC columns was washed using 10 mL of washing buffer (NaCl 2.5% *w*/*v*, NaHCO_3_ 0.5% *w*/*v*, Tween^®^ 20 0.01% *w*/*v*) and 10 mL of distilled water at a flow rate of 1/2 drops per second. Then OTA was eluted using methanol (1.5 mL) in a 4 mL screw-cap silanized vial. The evaporation of the eluted extracts was performed using a stream of air at ca 50 °C. Dried residues were redissolved in 500 μL of a mixture acetonitrile/water/acetic acid 99:99:2 (*v*/*v*/*v*). For the HPLC analyses, an aliquot (100 μL) of the solution was injected into the chromatographic apparatus Agilent 1260 Series chromatographic system (Agilent Technologies, Palo Alto, CA, USA), equipped with a fluorometric detector (model G1321B, ʎ_ex_ = 333 nm and ʎ_em_ = 460 nm). The chromatographic conditions were as follows: analytical column was a Zorbax SB-C18 (5 μm, 4.6 × 150 mm; Agilent Technologies), mobile phase was a mixture acetonitrile/water/acetic acid 99:99:2 (*v*/*v*/*v*), flow rate of 1 mL/min. Under these conditions, OTA retention time was *t* = 6.2 min and the limit of detection (signal-to-noise ratio of 3:1) and limit of quantification (signal-to-noise ratio of 10:1) of the method were 0.06 and 0.20 μg/kg, respectively.

### 4.7. In-House Validation of the Optimized FPIAs as Quantitative Method

LODs of the FPIA were calculated from the mean FP signal of representative blank samples (*n* = 10) subtracting three standard deviations of the mean signal [50]. LOQs were calculated by measuring the lowest amount of OTA, quantitatively determined by the calibration curve within the FPIA linearity range. For recovery experiments, blank samples were spiked in triplicate with OTA at levels of 2, 5 and 8 μg/kg. In order to allow solvent evaporation prior to extraction and analysis by both FPIA and HPLC method, spiked samples were left overnight at room temperature. A total of 20 samples of rye and 20 samples of rye crispbread artificially contaminated with OTA at different contamination levels were analysed for comparison of results by both FPIA and HPLC analysis.

### 4.8. In-House Validation of the Optimized FPIAs as Screening Method According to Regulation 519/2014/EU 

For the single laboratory validation, experiments were carried out according to a 5-day nested design, under repeatability conditions, resulting in 4 independent analyses per day, and twenty measurements in total, for blank and artificially contaminated rye samples at screening target concentration (STC) of OTA. The STC was the EU maximum permitted level of OTA in rye (i.e., 5 μg/kg). In the collaborative trial, each laboratory had to analyse 20 samples, 10 blank samples and 10 spiked samples at STC of OTA. The fitness of purpose of screening method was determined by evaluating the cut-off level and rate of false suspect results according to Regulation 519/2014/EU, as following Equation (1):

Cut-off = R_STC_ − *t*-value_(0.05)_ × SD_STC_ (1)

where the R_STC_ is the mean value of OTA content calculated from all 20 experiments carried out on artificially contaminated rye samples at STC of OTA in the single laboratory validation and the small scale collaborative trail; *t*-value_(0.05)_ is the one tailed *t*-value for a rate of false negative results of 5%; the SD_STC_, from the artificially contaminated rye samples at STC of OTA, is the corresponding standard deviation of intermediate precision, estimated by Nested ANOVA (*p*-value= 0.05) in the single-laboratory validation; whereas in the collaborative trial, it is the corresponding standard deviation of reproducibility, calculated according to the AOAC guidelines for collaborative studies [51].

The rate of false suspect results was obtained by calculating the *t*-value as the following Equation (2):

*t*-value = (cut-off − mean_neg_)/SD_neg_ (2)

where mean_neg_ is the mean value of OTA content obtained from all 20 experiments performed on blank rye samples in both validation studies; cut-off is the cut-off level established above; SD_neg_ is the corresponding standard deviation of intermediate precision of blank samples in the single-laboratory validation and the corresponding standard deviation of reproducibility of the blank samples in the collaborative trial. The values of standard deviation used for the calculation of *t*-value were obtained as reported above. From the obtained *t*-value, the rate of false suspect results for a one-tailed distribution was calculated as reported in Section 4.9. Concerning the small-scale collaborative trial, the precision was expressed in terms of RSD_r_ and RSD_R_. These values were calculated according to AOAC guidelines for collaborative studies [51].

### 4.9. Statistical Analysis

Sigmoidal fits of FPIA data were achieved by means of the unweighted least-square method using Origin version 6.0 (OriginLab Corporation, Northampton, MA, USA). The sigmoidal fit used the logistic equation y = A_2_ + [A_1_ − A_2_/1 + (x/x_0_)^P^], where A_1_ and A_2_ represent the initial (left horizontal asymptote) and the final value (right horizontal asymptote), respectively, x_0_ the centre (inflection point), and P the power. The parallelism and position statistical tests were used to compare linear regression curves [52]. For recovery experiments, Bartlett’s test and one-way ANOVA (*p*-value = 0.05) were applied in order to assess the homogeneities of variances and means respectively, among the spiking levels of contamination (*n* = 3). The statistical assessment of results from the single laboratory validation experiments was done by nested ANOVA (*p*-value = 0.05) using the software package MINITAB^TM^ Statistical Software for Windows, version 14 (Minitab, State College, PA, USA). The rate of false suspect results corresponding to the calculated *t*-value with one-tailed distribution was calculated using Student’s T Distribution (sheet function “TDIST”).

## Figures and Tables

**Table 1:** OTA average recoveries and relative standard deviations from spiked rye and rye crispbread obtained by FPIA and HPLC reference method.

Matrix	Spiking Level (μg/kg)	FPIA		HPLC	
		Recovery	RSD ^a^ (%)	Recovery	RSD ^a^ (%)
Rye	2	86	2	99	3
	5	84	2	98	2
	8	89	4	100	3
	**Overall average**	**86**	**3**	**99**	**3**
Rye crispbread	2	99	6	91	1
	5	86	2	96	6
	8	100	2	97	1
	**Overall average**	**95**	**3**	**94**	**3**

**^a^** RSD, relative standard deviation (*n* = 3 replicates).

**Table 2:** Statistical assessment of single-laboratory validation over 5 different days and small-scale collaborative trial of the FPIA for the determination of OTA in rye performed with artificially contaminated rye samples at a screening target concentration of 5 μg/kg and blank rye samples (with OTA content lower than LOQ_HPLC_). Cut-off level and rate of false suspect results were calculated according to Regulation 519/2014/EU.

Validation	Statistical Assessment	Blank	STC ^a^ (5 μg/kg)
**Single laboratory validation**	Mean value ^b^ (μg/kg)	1.1	4.3
	RSD_r_^c^ (%)	13	10
	RSD_RI_^d^ (%)	19	10
	Cut-off level		3.6
	Rate of false suspect results (%)	<0.1	
**Small scale collaborative trial**	Mean value ^b^ (μg/kg)	0.9	4.8
	RSD_r_^e^ (%)	16	9
	RSD_R_^f^ (%)	22	10
	Cut-off level		4.0
	Rate of false suspect results (%)	<0.1	

^a^ STC, screening target concentration. ^b^ The mean value (μg/kg) of the total content of OTA (*n* = 20 replicates). ^c^ RSD_r_, relative standard deviation of the repeatability obtained in the single-laboratory validation. ^d^ RSD_RI_, relative standard deviation (intermediate precision) obtained in the single-laboratory validation. ^e^ RSD_r_, relative standard deviation of repeatability (*n* = 10 replicates for each laboratory). ^f^ RSD_R_, relative standard deviation of reproducibility (*n* = 10 replicates for each laboratory).
